# Clinical and Radiological Improvement Following Ozone Disc Nucleolysis: A Case Report

**DOI:** 10.7759/cureus.1162

**Published:** 2017-04-13

**Authors:** Sharad Ghatge, Pranav D Modi, Dhaval B Modi

**Affiliations:** 1 Interventional Neuro-Radiology, Bombay Hospital and Medical Research Centre, Mumbai; 2 Trainee Research Assistant, Clinsearch Healthcare Solutions, Thane, Maharashtra; Dept. of Internal Medicine, K.J. Somaiya Hospital and Medical Research Centre, Mumbai; 3 Interventional Neuro Radiology, Bombay Hospital Institute of Medical Sciences and Research, Mumbai

**Keywords:** disc herniation, ozone, minimally invasive spine surgery, interventional neuroradiology, back pain, prolapsed intervertebral disc, sciatica, slipped disc

## Abstract

The results of traditional open surgery for herniated intervertebral disc are often limited by complications and failed back surgery syndrome (FBSS). Over the past few decades, a considerable amount of research has been done in the field of minimally invasive procedures as a treatment option for herniated intervertebral disc disease. Ozone disc nucleolysis is one such procedure which has results equal to or better than traditional surgery with virtually no complications. A 27-year-old post-partum female presented to the clinic with acute onset of severe lower back pain radiating to the left lower limb for one month. The pain started suddenly during labor and gradually increased over a period of few weeks post-partum. A magnetic resonance imaging (MRI) scan showed a large herniated disc bulge at the L4-L5 level causing severe radiculopathy. There was no bladder or bowel involvement. The patient was managed conservatively for three weeks. However, she failed to show any signs of improvement. She opted to undergo ozone disc nucleolysis under local anaesthesia. She showed significant improvement immediately after the procedure and there was further improvement in symptoms over a period of six weeks. Post-procedure follow-up at three months and six months showed significant improvement on the visual analogue scale (VAS), which was used to measure pain intensity and pain affect, along with the Oswestry Disability Index (ODI), which was used to measure the degree of disability due to the lower back pain.

Her VAS score improved from nine to two at three months and finally to one at six months, whereas the ODI score improved significantly from 46 to 10 at three months and eventually to four at six months. Ozone disc nucleolysis is an efficacious, safe, durable, and cost-effective treatment option for mild to moderate cases of herniated intervertebral disc which are resistant to conservative management. However, randomized control trials are required to build a long-term database regarding the efficacy and durability of ozone disc nucleolysis as compared to other minimally invasive procedures and surgery. We strongly believe that the availability of long-term data on ozone disc nucleolysis would make it a more acceptable form of treatment for disc herniation as compared to traditional surgery.

## Introduction

Back pain caused by intervertebral disc herniation is one of the most common symptoms for which a patient seeks medical treatment [1]. About 85% of the global population gets affected by intervertebral disc herniation at least once in their lifetime [1]. The first line of treatment is conservative management. It involves painkillers, bed rest, traction, and physiotherapy. However, about 40% of the patients do not get relief with conservative management. Previously, the only option available for treatment was open surgery. However, even successfully performed procedures sometimes result in complications and unsatisfactory outcomes [2]. The high number of failures associated with traditional surgery provided an impetus for the evolution of alternative minimally invasive percutaneous spine surgery techniques. Ozone disc nucleolysis is one of these emerging techniques which is effective in the treatment of intervertebral disc herniation and promises to give quick relief from lower back pain in these patients. Patients with mild to moderate disc disease who fail to respond to conservative management are good candidates for ozone disc nucleolysis. The current treatment options work on the principle of pain relief in disc herniation by reducing intra-discal pressure. Besides reducing the mechanical intra-discal pressure and correcting the ischemia and venous stasis, ozone disc nucleolysis also has an anti-inflammatory and immunomodulatory action [3]. This case report focuses on the management of a patient with a spinal disc herniation using ozone disc nucleolysis.

## Case presentation

A 27-year-old post-partum female presented to the clinic with severe lower back pain radiating to the left lower limb for one month. The visual analog scale (VAS) was used to measure pain intensity and pain affect and the Oswestry Disability Index (ODI) was used to measure the degree of disability due to lower back pain [4-5]. The patient's initial scores on the VAS and ODI scales were 9 and 46 respectively.

The lower back pain developed suddenly during labor. It was initially masked by labor pain and was unrecognised for a few days post-partum. The pain gradually increased over a few weeks and radiated to the dorsum of her left foot. She was given a trial of conservative management for three weeks following which she failed to show any signs of improvement. She had no history of lifting heavy weights, previous surgery, injuries to the back, fever, weight loss, abnormalities of bowel or bladder movements, weakness or loss of coordination of upper or lower limbs.

On examination, vital parameters were within normal limits. Her gait was slow, limited by lower back pain radiating to the dorsum of her left foot. Inspection of the lower back revealed normal anterior curvature of the lumbosacral spine without overlying skin abnormalities. Her range of motion was limited in a forward flexion. There was moderate tenderness present over the lower back. Straight leg raise (SLR) was 20 on the left side. This test is done with the patient in supine position. The examiner lifts one leg upward keeping the knee fully extended. If the patient experiences sciatic pain during the leg raise, the test is positive and suggests herniated disc as a possible cause of pain. Power in the left extensor hallucis longus (EHL) was 3/5 and the left ankle reflex was absent. All sensations were intact. Peripheral pulses were well felt bilaterally in both lower limbs. A systemic examination did not reveal any significant abnormality.

**Figure 1:**
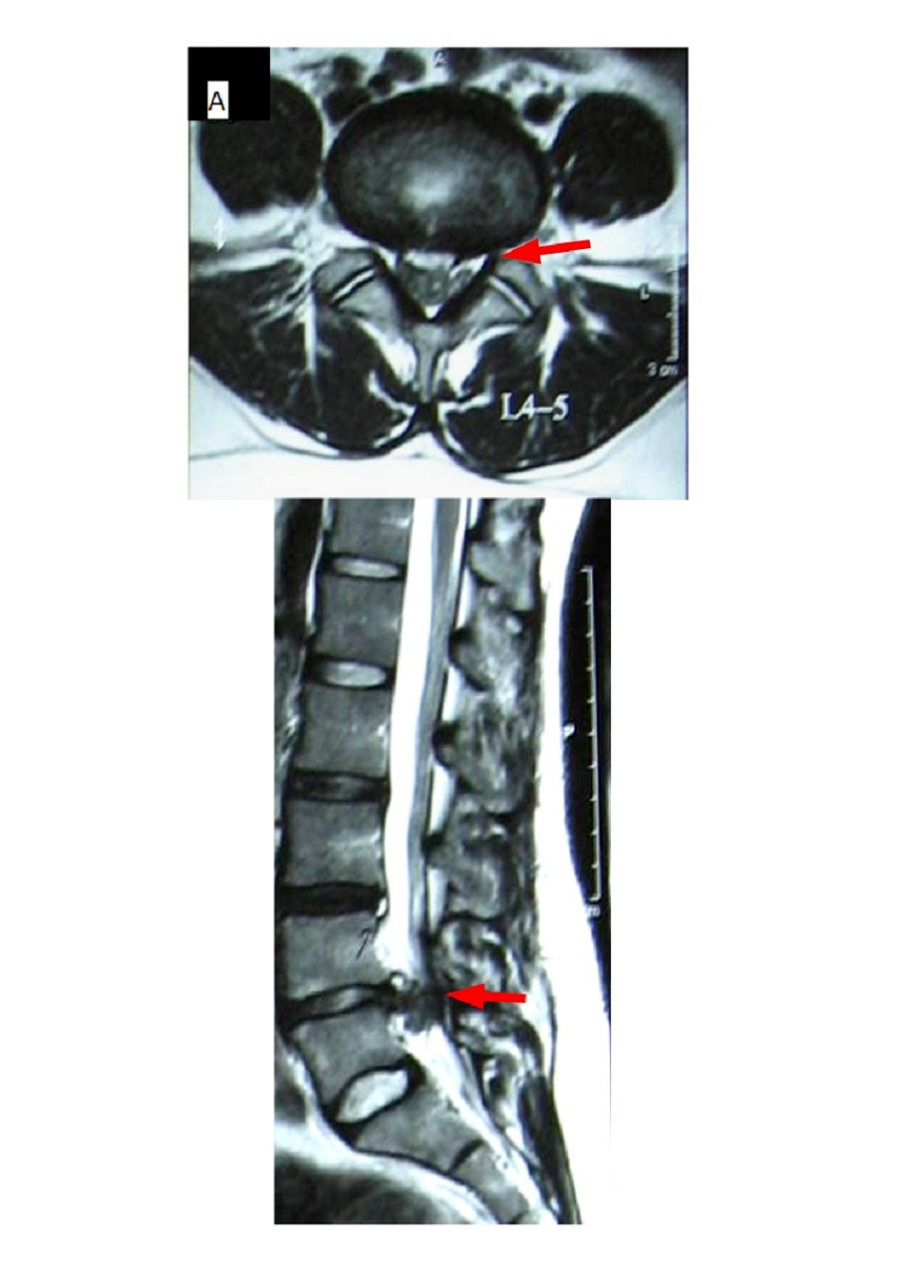
Axial and Sagittal T2W Images Pre-Procedure Large postero-central disc protrusion at the L4-L5 level.

A magnetic resonance imaging (MRI) scan at this point showed a large herniated disc bulge at the level of L4-L5, causing a large radiculopathy (Figure 1).

The patient was then given a trial of conservative management in the form of bed rest, physiotherapy, and analgesics. However, with conservative management, the patient did not show improvement and decided to undergo ozone disc nucleolysis.

The procedure was done under local anaesthesia in the catheterization laboratory on the Siemens rubber tired gantry (RTG) single-plane digital subtraction angiography (DSA) suite. With the patient in prone position, the C-arm was rotated such that the lateral one-third of the disc remained free from the lateral margin of the facet joint. Under local anaesthesia, a 22 gauge 17-cm long needle was introduced percutaneously via paraspinal approach into the disc via the transforaminal route. The intradiscal position of the needle was confirmed in the antero-posterior and lateral view. The ozonator was used to generate ozone on site by partly converting medical grade oxygen. 10 ml of ozone-oxygen mixture (concentration of 30 micrograms per ml) was injected intradiscally and in the periganglionic epidural space. Additionally, a mixture of local anaesthetic agent lidocaine 2%, hydrocortisone 100 mg, triamcenolone 45 mg, and hyaluronidase 1500 IU was also injected in the periganglionic epidural space.

Post-procedure, the patient was advised to remain in supine position for one hour, following which she was gradually mobilised and advised light activity for the following three weeks. She showed significant improvement immediately after the procedure and there was a further improvement in symptoms over the next six weeks.

**Figure 2:**
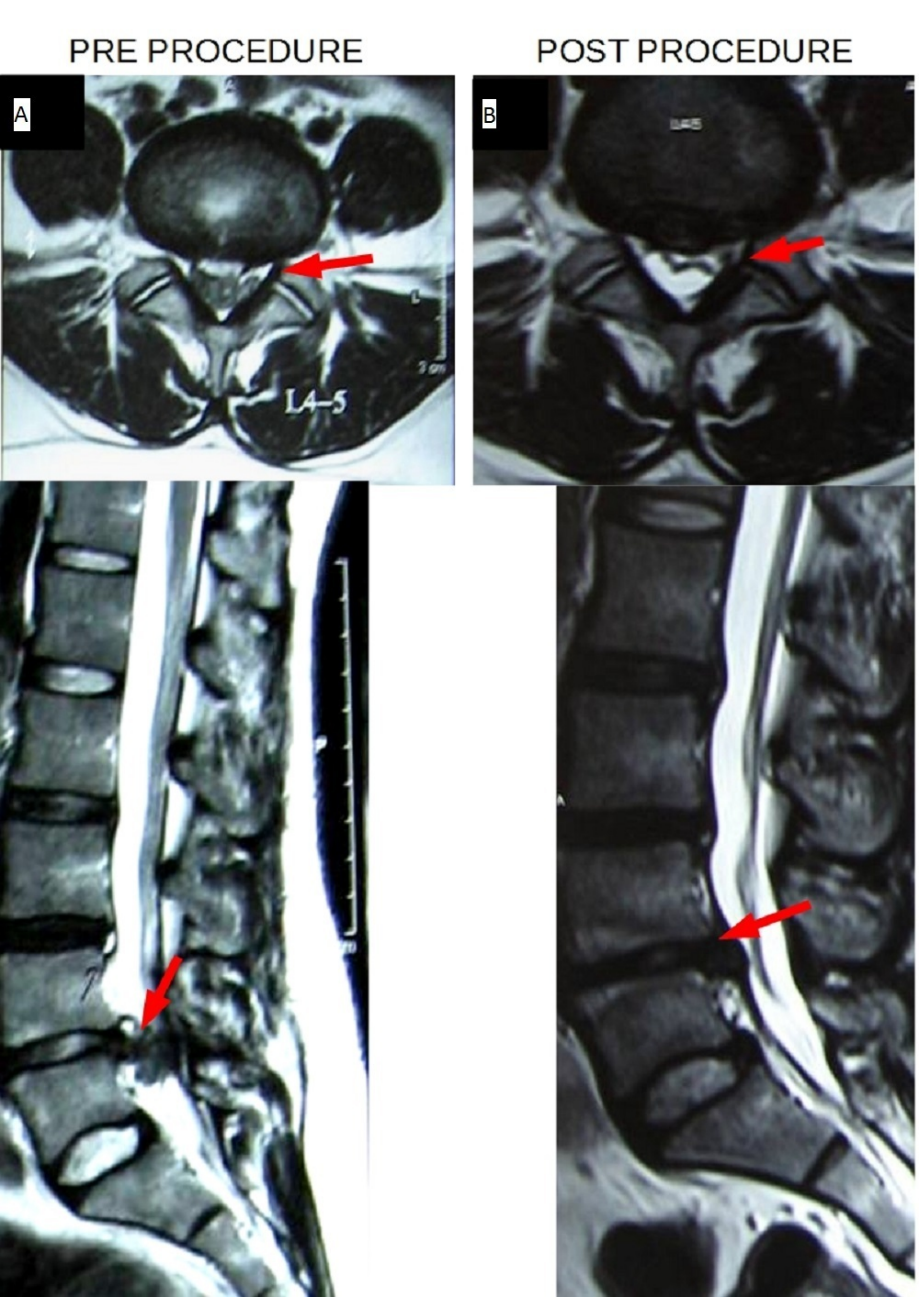
Pre-Procedure (A) vs. Post-Procedure (B) Axial and Sagittal T2W Images Large postero-central disc protrusion at the L4-L5 level (A) which showed significant resolution post ozone disc nucleolysis (B).

Post-procedure follow-up at three months revealed a VAS score of two and an ODI score of 10. The axial and sagittal T2-weighted (T2W) images on the MRI scan at three months showed significant resolution of the large postero-central disc protrusion at the level of L4-L5 following ozone disc nucleolysis (Figure 2).

Post-procedure follow-up at six months revealed a VAS score of 1 and an ODI score of 4. The patient was counselled to avoid strenuous activity for six months. She was also advised physiotherapy to strengthen core muscles. There were no complications following the treatment.

The MacNab criteria was used to determine the satisfaction and well-being of the patient post procedure [6]. Her outcome on MacNab was in the excellent category.

## Discussion

Minimally invasive procedures such as ozone disc nucleolysis have emerged for the treatment of disc herniation in recent times. Ozone disc nucleolysis has many advantages as it is a fast, cost-effective and efficacious procedure with minimal complications. Ozone disc nucleolysis has become a popular choice in recent years for treatment of large herniated discs or for acute or worsening lower limb weakness as seen in cauda equina syndrome [7].

The mechanism by which ozone disc nucleolysis brings pain relief is unique. A mixture of ozone and oxygen (O_3_-O_2_) is used for the procedure where the ozone reaches a maximum of five percent of the total mixture. This causes an inactivation and inhibition of the release of proteolytic enzymes and of endogenous reactive oxygen species (ROS) [8]. Ozone therapy also stimulates proliferation of chondrocytes and fibroblasts with increased synthesis of the matrix and articular cartilage. Ozone acts on erythrocytes by increasing the concentration of 2,3-diphosphoglycerate (2,3-DPG) which causes the left shift of the Hb-O_2_ curve and increased peripheral tissue oxygenation [8]. Ozone inhibits the synthesis and release of proinflammatory substances which decreases pain and edema [3,8]. It releases immunosuppressive cytokines. It also has an effect on pain pathways through the activation of the descending antinociceptive system and release of endorphins, which block transmission of the noxious signal to the thalamus and cortex [9].

Minimally invasive techniques have progressed extensively over the last few decades and along with ozone disc nucleolysis, there are procedures like chemonucleolysis and percutaneous discectomy which can be offered to patients.

We propose a three-step treatment protocol for the treatment of herniated intervertebral disc. Step 1 involves conservative management for three weeks, Step 2 involves ozone disc nucleolysis and Step 3 involves surgery. Recovery is also seen in three stages. Stage 1 includes the first two weeks (post-procedure) that shows complete or partial relief. Stage 2 is the intermediate stage (from two to six weeks post-procedure) in which pain may recur or there is no further recovery from Stage 1. Stage 3 is the consolidation phase (six weeks post-procedure) in which there is further pain relief and stable recovery.

Other minimally invasive techniques like chemonucleolysis with chymopapain, laser discectomy, intradiscal electrothermal therapy, and DiscoGel® are also available for the treatment of disc herniation. However, ozone disc nucleolysis has results comparable with surgical microdiscectomy and DiscoGel® chemolnucleolysis along with the best cost-benefit ratio. The likelihood of complications is very low with ozone disc nucleolysis and it has the lowest complication rate ( < 0.1%) among all other minimally invasive techniques [10].

Interventional radiologists need to adopt minimally invasive techniques and gain more experience in performing such procedures. It is necessary for training institutes to introduce these techniques as they can provide improved outcomes for patients. As of now, ozone disc nucleolysis is available at limited centres in India. However, the availability is expected to increase in view of the promising results of our case series.

## Conclusions

Ozone disc nucleolysis is an efficacious, safe, durable, and cost-effective treatment option for the treatment of herniated intervertebral disc. However, randomized control trials are required to build a long-term database regarding the efficacy and durability of ozone disc nucleolysis as compared to the other minimally invasive procedures and surgery. We strongly believe that with the availability of long-term data, techniques like ozone disc nucleolysis can be recommended as an effective alternative to traditional surgery for disc herniation.

## Appendices

The visual analog scale (VAS) was used to measure pain intensity and pain affect. It has a line measuring 100 mm with extremes labelled as ‘no pain’ and ‘pain as bad as it could be’. The patient is asked to put a mark at a point on the line indicating current pain intensity. The score is obtained by measuring the distance between the mark and the 'no pain' end. Numbers 0-10 are provided along the scale for guidance and the score can be reported as no pain (0), mild (1-3), moderate (4-6), severe (7-9), pain as bad as it could be (10) [4].

The Oswestry Disability Index (ODI) was used to measure the degree of disability due to lower back pain. The patient fills a questionnaire which has questions concerning intensity of pain, lifting, ability to care for oneself, ability to walk, ability to sit, sexual function, ability to stand, social life, sleep quality, and ability to travel. The scores for all questions answered are summed and reported as minimal disability (0-20), moderate disability (21-40), severe disability (41-60), crippling back pain (61-80), and bed-ridden (81-100) [5].

MacNab cirteria was used to determine post-procedure patient satisfaction and well-being. The patient selects one out of four grades which are excellent (no pain, no restriction of mobility, return to normal work and level of activity), good (occasional nonradicular pain, relief of presenting symptoms, able to return to modified work), fair (some improved functional capacity, still handicapped and/or unemployed), or poor (continued objective symptoms of root involvement, additional operative intervention needed at index level irrespective of length of postoperative follow-up) [6]. 
